# Codivir suppresses SARS-Cov-2 viral replication and stabilizes clinical outcome: In vitro and Phase I clinical trial results

**DOI:** 10.15190/d.2022.17

**Published:** 2022-12-31

**Authors:** Yotam Kolben, Eynat Finkelshtein, Esmira Naftali, Ariel Kenig, Asa Kessler, Florentino Cardoso, Nadya Lisovoder, Asaf Schwartz, Daniel Elbirt, Shlomo Maayan, Yaron Ilan

**Affiliations:** ^1^Faculty of Medicine, Hebrew University Hadassah Medical Center, and the Department of Medicine, Hadassah Medical Center Jerusalem, Israel; ^2^Code Pharma, The Netherlands; ^3^Hospital is Casa de Saúde - Vera Cruz, Brazil; ^4^Clinical Immunology, Allergy and AIDS Center Kaplan Medical Center, Affiliated with Hadassah-Hebrew University Medical School Jerusalem, Rehovot, Israel; ^5^Division of infectious diseases, Barzilai University Medical Center, Ashkelon, Israel

**Keywords:** COVID-19, SARS-CoV-2, anti-viral therapy, Codivir.

## Abstract

BACKGROUND: Treatment of severe acute respiratory distress syndrome coronavirus 2 (SARS-CoV-2) remains a significant challenge in the face of increased worldwide morbidity and mortality. The acute illness caused by SARS-CoV-2 is initiated by a viral phase, followed by an inflammatory phase. Numerous anti-inflammatory and anti-viral therapies, with a relatively minor clinical effect, have been applied. Developing a safe and efficient direct anti-viral treatment is essential as it can block disease progression before significant complications ensue and potentially prevent transmission. AIM: The present phase 1 study aimed to determine the safety of Codivir, a newly developed anti-viral agent, and to preliminarily assess its anti-viral activity in patients infected by COVID-19. METHODS: In vitro studies were conducted to determine the direct anti-viral effect of Codivir using an immunofluorescence-based assay and to assess its cytotoxic effect by tetrazolium assay (MTT). In a phase I clinical trial, Codivir was administered for ten days in 12 patients who were followed for its safety. Patients were followed for clinical manifestations during administration. Sequential nasal viral PCR titers (Cycle Threshold, CT) were determined preceding and during treatment. RESULTS: In vitro, Codivir showed activity against SARS-CoV-2 with 90% viral replication suppression and minimal cytotoxicity. The anti-viral activity was demonstrated at the early stages of infection, post-entry of the virus in the cell. Codivir was safe in all 12 patients in phase I clinical trial and significantly suppressed viral replication in 5/7 fully assessed patients, with an anti-viral effect noted as early as three days. SUMMARY: The present study's data support the safety of Codivir administration in humans and suggest its significant anti-COVID-19 effect. These results support the testing of the drug in more extensive controlled trials in patients with SARS-CoV-2.

## INTRODUCTION

The coronavirus disease 19 (COVID-19) pandemic has spread rapidly worldwide. The spread of the severe acute respiratory distress syndrome coronavirus 2 (SARS-CoV-2) led to a pandemic that, as of November 2022, had caused more than 638 million cases, 6.62 million confirmed deaths, and left many more with significant sequelae^1-3^. In contrast to the rapid development of efficient anti-SARS-CoV-2 vaccines, therapies for COVID-19 were less successful^4-6^.

The pathogenesis of SARS-CoV-2 infection is attributed to a complex interplay between the virus and host immune response associated with the activation of multiple inflammatory pathways leading to hyperinflammation and cytokine storm^7,8^. Direct anti-viral agents and numerous anti-inflammatory therapies are applied to these patients^6,9-11^. Glucocorticoids -anti-IL-6 based drugs are some anti-inflammatory therapies showing some effects in patients with different degrees of severity of illness^9,12,13^. Developing a safe and efficient anti-viral treatment is vital as it can block disease progression before significant complications ensue and prevent transmission.

Codivir (Code Pharma, The Netherlands) is a 16 amino-acid synthetic peptide derived from the HIV-1 integrase. It was designed and developed as a therapeutic agent for HIV-infected patients, originally named integrase stimulatory (INS) peptide. This peptide promotes the integration of multiple HIV DNA copies into the host cells genomic DNA to the extent that it triggers the self-destruction of infected cells by apoptosis^14^. It exerts a direct -anti-SARS-CoV-2 effect via a different mechanism of action, not by apoptosis.

Here we report the results of an *in vitro* study and phase I clinical trial-using Codivir in patients infected with SARS-CoV-2.

## MATERIALS AND METHODS

### Codivir

Codivir (Code Pharma) is a chemically synthesized 16 amino-acid peptide, which was designed, based on the HIV-1 integrase, and exerts direct anti-viral properties.

### Pre-clinical study

#### a. Anti-viral Effect

*In vitro* studies (conducted by Virology Research Service, England^15^ were aimed to examine the anti-viral activity and cytotoxicity of Codivir against SARS-CoV-2. Eight dilutions of Codivir were explored along with infection or 2 hours post-infection. Viruses and peptides were left on the cells for 24 hours. A standard tetrazolium assay (MTT) assay determined cytotoxicity. As controls, uninfected treated cells, infected untreated cells, and infected cells treated with a positive control drug (remdesivir) were used.

Cells (Vero E6) were seeded in complete media at 8,000 cells/100 µl/well and were incubated at 37°C, 5% CO_2 _for 24 hours. SARS-CoV-2/England/2/2020 virus (the Biodefense and Emerging Infections Research Resources Repository, BEI Resources, England) at 0.002 multiplicity of infection (MOI) was added to cells.

75 µM of Codivir peptide was added, immediately or after 2 hours, to wells in the sequential row, serially diluted (3-fold). As a control, 40 µM remdesivir was added to appropriate wells similarly. After 24 hours, one plate was used for cytotoxicity analysis and treated with MTT to determine cell viability. The other plate was stained with an anti-SARS-CoV-2 spike protein antibody (GeneTex, USA). The primary antibody was detected with an Alexa-488 conjugated secondary antibody (Life Technologies, India), and nuclei were stained with Hoechst stain. Images were acquired on an Opera Phenix confocal microscope (Perkin Elmer, USA), and the percentage of infection was calculated using Columbus software (infected cells/total cells x 100).

Normalized percentages of inhibition and normalized percentage of cytotoxicity were calculated. Effective concentration (EC)50 and toxic concentration (TC) 50 were extrapolated from curves representing the best fit (non-linear regression analysis, variable slop) of the logarithm of compound concentration vs. normalized percentage of inhibition, and cytotoxicity, respectively, using GraphPad Prism (version 9).

#### b. Stages of viral replication inhibition

To determine the stages of replication inhibited by Codivir peptide, 16,000 cells/100 µl/well were either infected with 0.05 MOI SARS-CoV-2 for 8h or with 0.002 MOI for 24h and then treated with three peptide concentrations (75µM 12.5µM and 4.17 µM). An immunofluorescence-based assay determined anti-viral activity at 8h or 24h. Cytotoxicity was determined using an MTT assay on uninfected cells with the same peptide concentrations. The assessment was performed as in the anti-viral effect study.

### Phase I Clinical trial

#### a. Ethical Considerations

The Research Ethics Committee approved the phase I study of the Faculty of Medicine of Jundiaí. NIH Clinical trials Gov number NCT04930861. The study was performed in Brazil, as Brazil was an epicenter of the COVID-19 pandemic, resulting in a high burden of COVID-19 patients and overflowed hospitals^16^. Therefore, after enrolling the first three patients, several amendments were made to facilitate recruitment and, more importantly, reduce the burden inflicted by the study patients on the local health system. The relevant authorities approved the amendments. All patients provided informed consent for the study.

#### b. Inclusion criteria

Hospitalized patients with moderate COVID-19, defined as a clinical or radiological diagnosis of pneumonia according to accepted criteria^17^, with oxygen saturation in room air >93% and <30 breaths per minute.

#### c. Study Population

Twelve patients (>18 years old) with mild to moderate COVID-19 were enrolled in a phase I clinical trial. The study was conducted at Vera Cruz medical center, São Paulo, Brazil. COVID-19 was diagnosed by a positive real-time polymerase chain reaction (RT-PCR) from a nasopharyngeal swab. The main inclusion criteria included evidence of pneumonia, oxygen saturation >93%, less than 30 breaths per minute, and not requiring oxygen support (defining moderate COVID-19 illness) with symptoms initiation less than 72 hours before enrollment. The symptoms' duration was later prolonged to 96 hours to increase the recruitment rate. None of the patients received anti-viral drugs, including Remdesivir, at the time of inclusion or afterward.

Patients suffering from severe COVID-19, defined as O2 saturation below 93% on room air or hemodynamic instability, were excluded. Likewise, mild cases not having pneumonia on chest X-ray (CXR) were excluded. In addition, pregnant or lactating women, patients with a significant acute or chronic medical condition, patients positive for human immunodeficiency virus (HIV), and patients with active hepatitis B virus (HBV) or hepatitis C virus (HCV) infection were excluded.

#### d. Study Design

After screening for inclusion and providing informed consent, patients were administered a ten-day course of Codivir 20 mg twice a day (BID) subcutaneously. Initially, patients were hospitalized at least until day 4 of treatment. However, after enrolling the first three patients, an amendment was approved to discharge patients six hours after the first subcutaneous dose with a subsequent twice-daily subcutaneous administration and follow-up by a physician or a study nurse at home until day 10.

The twice-daily assessment included vital signs, national early warning score 2 (NEWS2), and recording adverse events. In addition, blood samples for D-dimer, C-reactive protein (CRP), alanine transaminase (ALT), and aspartate aminotransferase (AST) were obtained daily. From patient #4 onward, nasal viral load (by computerized tomography, CT value) was obtained every 48 hours.

#### e. Endpoints

The study's primary objective was to determine the safety of Codivir in patients with moderate COVID-19. Therefore, the main follow-up parameter was the rate of serious adverse events (SAE). Adverse events were defined as SAE if resulting in patients' death, required hospital admission or prolonged current hospitalization, resulted in persistent disability, or were considered severe by the investigators.

Secondary outcomes for the initial evaluation of treatment efficacy included changes in viral load measured by PCR cycles (CT values, using AllplexTM 2019-nCoV Assay version 2.2; April 15th, 2021, Seegene for E gene amplification, according to the manufacturer's instruction on the sp2000rt instrument (Abbott, Ill)) and progress if any of NEWS2 score.

## RESULTS

### *In vitro* study: Codivir showed a potent anti-viral effect

**Figure 1:**
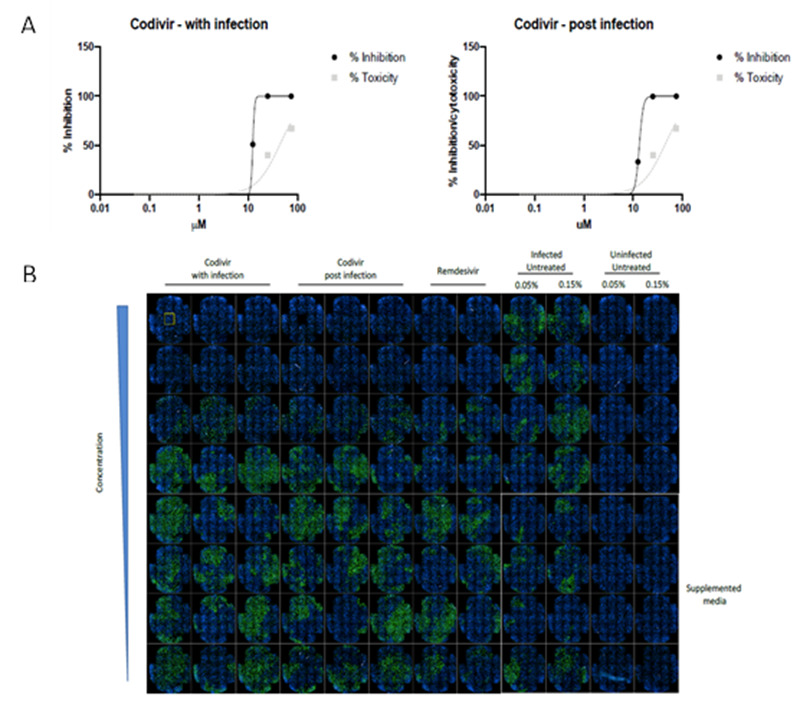
In-vitro anti-SARS-CoV-2 effect of Codivir A. Graphs displays the percentage of inhibition of SARS-CoV2 infection (black line) at different peptide concentrations. The x axes show compound dilutions (µM). The curves represent the best fit of the logarithm of compound dilution vs. the normalised percentage of inhibition (variable slope). Cytotoxicity is displayed in grey. B. Images of cells infected or uninfected with or without compound.Green – stain for SARS-CoV-2 spike protein, Blue – nuclei stain.

Inhibition of SARS-CoV-2 was observed in cells treated with Codivir, with EC50 of 12.48 µM (peptide administered at the same time as the virus) and 13.34 µM (peptide administered 2 hours post-infection) (Table 1 and Figure 1). EC90 for the same experimental conditions was 13.7 µM and 16.6 µM, respectively. Some cytotoxicity was observed at the highest concentrations tested, with a TC50 value of 43.48 µM and a TC90 value of 143.6 µM. The suppressor method SI50 was between 3.48 and 3.26, and the suppressor method SI90 was between 10.5 and 8.6.

**Table 1:** In-vitro analysis of effective and toxic concentration of Codivir Abbreviations: Effective concentration (EC); not determined (ND) (due to inability to extrapolate a curve from input values); selectivity index (SI); toxic concentration (TC).

Test article	EC50 (µM)	TC50 (µM)	SI50	EC90 (µM)	TC90 (µM)	SI90	EC99.9 (µM)	TC99.9 (µM)
Codivir – with infection	12.48	43.48	3.48	13.7	143.6	10.5	16.8	185.9
Codivir – post-infection	13.34	43.48	3.26	16.6	143.6	8.6	25.2	185.9
Remdesivir – with infection	1.28	ND		3.9	ND		4.4	ND

### *In vitro* Study: Codivir inhibits viral replication in early stages

**Figure 2:**
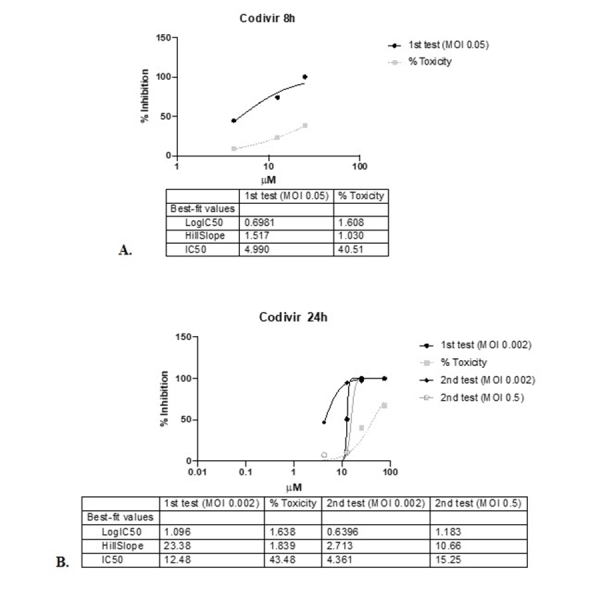
Codivir peptide exhibit inhibition of SARS-CoV-2 within 8 hours post infection, with an IC50 of 5µM Graph displays the percentage of inhibition of SARS-CoV2 infection (black line) at different peptide concentrations. The x axes show compound dilutions (µM). The curves represent the best fit of the logarithm of compound dilution vs. the normalised percentage of inhibition (variable slope). Cytotoxicity is displayed in grey. A. 8 hours. B. 24 hours. Also displays Figure 1’s results.

The anti-viral activity was already noted within eight hours, suggesting that the peptide already acts at the early stages of viral infection. Cytotoxicity test at 8h revealed the same pattern as at 24h (Figure 2).

### Clinical trial: Patients

Twelve patients, 7 females and 5 males, with a mean age of 45.4±11.3 years and a mean NEWS2 score of 1.83, were enrolled in the trial. Table 2 displays the baseline patients' characteristics.

**Table 2:** Baseline Patients' Characteristic

	N=12
Mean Age Age range	45.4± 11.3 23-60
Sex: male female	5 (42) 7 (58)
Race: white black	10 (83) 2 (17)
Medication: yes no	9 (75) 3 (25)
Weight	72.2 ±12.6
Height	165 ±12
BMI	26.3 ±2.2
Temp	36.5± 0.40
SBP	126± 23
DBP	81 ±12
Puls	82 ±14
Resp	18± 1.0

### Safety of Codivir

Codivir showed a high safety profile in all 12 patients. Patients were treated for 10 days. 11/12 patients completed 10 days of therapy using a twice-daily subcutaneous dose. Codivir was well tolerated, and no treatment-related major adverse events were recorded in any of the treated patients. Minor adverse events were non-significant.

### Anti-viral effect of Codivir

Seven patients were thoroughly evaluated in sequential nasal viral load (by PCR CT values). In 5 patients (no. 4, 6, 8,10,12), a potent and consistent anti-viral effect was noted during treatment with Codivir; In 2 patients (no. 5,9), the CT values obtained during follow-up were inconsistent with the expected change in CT seen in patients having an anti-viral response. Patient No. 8, stopped therapy after 3 days due to elevated liver enzymes, which appeared before treatment initiation; however, his viral load remained low at the end of the follow-up.

**Table 3 **shows the anti-viral effect of Codivir in treated patients as measured by PCR cycles (Cycle Threshold, CT). While all patients had positive qualitative PCR tests, quantitative data were available on 7 patients. As assessed by PCR, a decline in viral load was seen as early as 3 days from therapy.

**Table 3:** Effect of Codivir on viral load (PCR cycles) in patients infected with SARS-Cov-2

Patient No.	V1	V3	V5	V7	V10
4	23.2	28.4	31.63	30.31	36.32
5	19.11	24	Undetected	Undetected	28.64
6	20.74	22.34	28.57	32.03	Undetected
8	15.41	21.81	28.27	14.72	26.98
9	17	21.09	27.4	25.34	19.21
10	Undetected	14.39	21.79	31.15	30.05
12	16.51	14.82	20.41	26.25	36.23

### Clinical status of patients treated with Codivir

In 11 of the 12 patients, an improvement in the clinical condition and stabilization of the medical condition was noted.

A complete description of the clinical results is enclosed in the **Supplementary information**.

## DISCUSSION AND CONCLUSION

The present in vitro study data show that Codivir, a direct anti-viral agent, significantly suppressed viral replication in an *in vitro* model. Viral replication was inhibited at early stages and post-resenting the virus to the cell.

The phase I clinical trial data show that the drug has a high safety profile in humans and exerts a potent anti-viral effect in most (5/7) of the evaluable patients studied.

Codivir showed activity against SARS-CoV-2 at 24h post-infection in the pre-clinical study. 90% inhibition of infectivity was reached at concentrations only marginally higher than the EC50 value. At these concentrations, minimal cytotoxicity was observed, as reflected by higher selectivity index values. Codivir suppressed viral replication in all treated patients in the clinical trial, with an effect noted within three days.

The current two significant treatment venues for SARS-CoV-2 are anti-viral and immunomodulatory agents. These are based on the two phases of the disease. While several immunomodulatory agents, including glucocorticoids and anti-IL6, showed some efficacy, their long-term efficacy is limited^12,13,18-20^. The use of anti-viral antibodies showed moderate results^21,22^. Direct anti-viral therapy is a promising way to the management of these patients. Several anti-viral agents were being evaluated to treat SARS-CoV-2 infection^23,24^. Oseltamivir, favipiravir, umifenovir, lopinavir, remdesivir, hydroxychloroquine, chloroquine, azithromycin all failed to show a significant therapeutic effect. Newer anti-viral agents such as remdesivir showed some beneficial effects in subsets of patients^25^. Screening of a library of compounds containing approved RNA dependent RNA polymerase inhibitor drugs that were or are in use to treat other viruses, favipiravir, sofosbuvir, ribavirin, lopinavir, tenofovir, ritonavir, galidesivir, remdesivir, and molnupiravir, and their structural analogs were recently conducted to identify inhibitors of SARS-CoV-2. Extensive screening, molecular docking, and molecular dynamics show that several structural analogs have notable inhibitory effects on the virus. However, the limited available clinical data suggests that most currently approved anti-viral agents are effective in only subsets of patients^26^. Their clinical benefit in advanced cases is insignificant^27-32^.

The present study's data show that Codivir has a direct anti-viral effect *in vitro* and exerted a potent in vivo rapid anti-viral activity in most patients we studied. Codivir was well tolerated, and the treatment stabilized the clinical condition of infected patients as measured by clinical and laboratory parameters.

The data supports the concept of a two-phase disease. While the administration of a direct anti-viral agent can suppress the viral load, its effect on the immune phase may not be noticed within a short period. Early administration of the direct anti-viral drug may improve clinical outcomes and shorten the disease.

These preliminary results support further testing of Codivir in patients with SARS-CoV-2 using a larger cohort and a double-blind strategy to continue research on this promising drug.
